# Probiotic *Escherichia coli* Nissle 1917 Inhibits Leaky Gut by Enhancing Mucosal Integrity

**DOI:** 10.1371/journal.pone.0001308

**Published:** 2007-12-12

**Authors:** Sya N. Ukena, Anurag Singh, Ulrike Dringenberg, Regina Engelhardt, Ursula Seidler, Wiebke Hansen, André Bleich, Dunja Bruder, Anke Franzke, Gerhard Rogler, Sebastian Suerbaum, Jan Buer, Florian Gunzer, Astrid M. Westendorf

**Affiliations:** 1 Department of Mucosal Immunity, Helmholtz Centre for Infection Research, Braunschweig, Germany; 2 Department of Haematology, Haemostaseology and Oncology, Hannover Medical School, Hannover, Germany; 3 Department of Gastroenterology, Hepatology and Endocrinology, Hannover Medical School, Hannover, Germany; 4 Institute for Laboratory Animal Science and Central Animal Facility, Hannover Medical School, Hannover, Germany; 5 Immune Regulation Group, Helmholtz Centre for Infection Research, Braunschweig, Germany; 6 Department of Internal Medicine, Gastroenterological and Hepatology Unit, University Hospital of Zurich, Zurich, Switzerland; 7 Institute of Medical Microbiology, University Hospital Essen, Essen, Germany; 8 Faculty of Medicine Carl Gustav Carus, Institute for Medical Microbiology and Hygiene, Technology University (TU) Dresden, Dresden, Germany; The Research Institute for Children, United States of America

## Abstract

**Background:**

Probiotics are proposed to positively modulate the intestinal epithelial barrier formed by intestinal epithelial cells (IECs) and intercellular junctions. Disruption of this border alters paracellular permeability and is a key mechanism for the development of enteric infections and inflammatory bowel diseases (IBDs).

**Methodology and Principal Findings:**

To study the *in vivo* effect of probiotic *Escherichia coli* Nissle 1917 (EcN) on the stabilization of the intestinal barrier under healthy conditions, germfree mice were colonized with EcN or K12 *E. coli* strain MG1655. IECs were isolated and analyzed for gene and protein expression of the tight junction molecules ZO-1 and ZO-2. Then, in order to analyze beneficial effects of EcN under inflammatory conditions, the probiotic was orally administered to BALB/c mice with acute dextran sodium sulfate (DSS) induced colitis. Colonization of gnotobiotic mice with EcN resulted in an up-regulation of ZO-1 in IECs at both mRNA and protein levels. EcN administration to DSS-treated mice reduced the loss of body weight and colon shortening. In addition, infiltration of the colon with leukocytes was ameliorated in EcN inoculated mice. Acute DSS colitis did not result in an anion secretory defect, but abrogated the sodium absorptive function of the mucosa. Additionally, intestinal barrier function was severely affected as evidenced by a strong increase in the mucosal uptake of Evans blue *in vivo*. Concomitant administration of EcN to DSS treated animals resulted in a significant protection against intestinal barrier dysfunction and IECs isolated from these mice exhibited a more pronounced expression of ZO-1.

**Conclusion and Significance:**

This study convincingly demonstrates that probiotic EcN is able to mediate up-regulation of ZO-1 expression in murine IECs and confer protection from the DSS colitis-associated increase in mucosal permeability to luminal substances.

## Introduction

The epithelial layer of the gastrointestinal tract serves as one of the primary interfaces with the outside world. The mucosal surface of the intestinal epithelium is in constant contact with abundant populations of microbes and their metabolites. The intestinal barrier formed by the epithelial cells and the junctional complex, consisting of tight junctions (TJ), adherens junctions, gap junctions and desmosomes, excludes the majority of these microbes and their metabolites from access to the subepithelial cells. Its effectiveness and stability are ensured by the junctional complex [1]. Compromising the integrity of this barrier can promote manifestation of enteric infections and is a key feature of IBDs like Crohn's disease (CD) and ulcerative colitis (UC). Intestinal permeability was also found to be increased in HIV infection [2], and diarrhea is one of the most predominant symptoms of HIV-infected patients. The diarrhea of these patients is mainly due to infections with enteropathogens. However, in a number of HIV patients with gastrointestinal complaints no enteropathogen can be identified [3], [4]. The role of a ‘leaky gut’ in the pathogenesis of gastrointestinal diseases is increasingly recognized. Consequently, reduction of the increased permeability is an interesting target for improvement of the clinical status of gastrointestinal diseases. Tight junctions are intricate macromolecular protein structures located at the most apical regions of the junctional complex, sealing the spaces between the IECs. The first junction-associated protein identified was zonula occludens 1 (ZO-1), with a molecular mass between ∼210–225 kDa [5]. This molecule constitutes the structural link between the cytoskeleton and the tight junction by binding to both actin filaments and the TJ protein occludin [6]. Reorganization of TJ proteins like ZO-1, triggered by cytokines produced secondary to the inflammatory processes in IBDs, results in increased intestinal permeability [7], [8]. ZO-2 is another TJ associated protein that forms a complex together with ZO-1 [9] and has recently been shown to be up-regulated by EcN *in vitro* [10].

Probiotics are defined as live microorganisms which, when administered in adequate amounts, confer health benefits to the host [11]. The use of such microorganisms as novel therapeutic agents and as an alternative to standard medication in gastrointestinal diseases is promising, although their mechanism of action is still under investigation. EcN has evolved into one of the best characterized probiotics, and its therapeutic efficacy and safety have convincingly been proven [12]–[16]. A potential mechanism by which probiotics may exhibit their beneficial activities is modulation of the epithelial barrier function [17], [18]. This hypothesis is also supported by a recent study demonstrating that probiotic *Streptococcus thermophilus* and *Lactobacillus acidophilus* can prevent invasion of enteroinvasive *E. coli* and enhance intestinal epithelial barrier function by amplifying phosphorylation of occludin and ZO-1 *in vitro* [19].

Driven by mounting evidence affirming the beneficial effects of probiotics on the intestinal epithelial barrier and the already well-documented therapeutic efficacy of EcN, we set out to investigate the impact of EcN on the intestinal epithelial barrier function *in vivo*.

## Materials and Methods

### Mice

All mice used in this study were 6–8 weeks old females. Conventional BALB/c mice were obtained from Harlan (Borchen, Germany). Gnotobiotic BALB/c mice were obtained from colonies maintained germfree at the Central Animal Facility of Hannover Medical School, as described previously [20]. The animal experiments reported here were conducted in accordance with the German Animal Welfare Law and with the European Communities Council Directive 86/609/EEC for the protection of animals used for experimental purposes. All experiments were approved by the local institutional animal care and research advisory committee and authorized by the district authority of Braunschweig and Hannover.

### Bacterial colonization of gnotobiotic BALB/c mice

EcN or *E. coli* MG1655 was freshly grown to an OD_600_ = 1 (∼0.8–1.2×10^9^ CFU/ml) from an overnight culture diluted 1:500 in LB media [21]. Bacteria were collected by centrifugation (1 ml, 3 min at 1000×g). The resulting pellet was redissolved in 200 µl sterile PBS and administered by oral gavage. Application was repeated two days later. After 6 days of colonization, fecal CFU were determined by plate count from pooled stool samples and were calculated per gram feces. The animals grew comparable numbers of both bacterial strains in all experiments averaging 5.8×10^9^ CFU/g feces with EcN and 5.1×10^9^ CFU/g feces with *E. coli* MG1655. Feces of mice that had received PBS remained sterile.

### Induction of colitis

**Figure 1:**
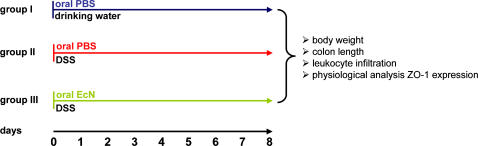
Experimental design of the DSS colitis model. Acute colitis was induced by administration of 4–6% DSS to drinking water (DSS). (A) Group I was orally treated with PBS twice daily. Group II was given 4–6% DSS in drinking water and orally treated with PBS twice daily. Group III received 1.5–2×10^8^ CFU EcN two times a day by oral application in combination with 4–6% DSS in drinking water (DSS+EcN).

Acute colitis was induced in BALB/c mice by addition of 4–6% dextran sodium sulfate (DSS) (MP Biomedicals, Eschwege, Germany) to drinking water for a period of 8 days, according to a protocol recently described by Grabig et al. [22]. Animals were separated into the following groups: Group I was treated orally with PBS two times a day. Group II received drinking water with 4–6% DSS and was treated orally with PBS twice daily. Group III also received drinking water with 4–6% DSS and was given 1.5–2×10^8^ CFU (Mutaflor mite, Ardeypharm, Germany) EcN twice daily by oral application (DSS+EcN) (Figure 1). Colitis induction indicated by weight loss of the mice was monitored by comparing the body weight upon DSS treatment to the initial body weight of the respective animals.

### Isolation of IECs

IECs were isolated as described elsewhere [23]. Briefly, the small and/or large intestine were isolated, rinsed with PBS and opened longitudinally. Mucus was removed by treatment with DTT for 15 min at 37°C on a shaker. Having washed the mucosa in PBS, it was placed in HBSS/1.5 mM EDTA and tumbled for 10 min at 37°C. The supernatant was collected and the remaining mucosa was vortexed in PBS. This supernatant was also collected. Pooled IECs were centrifuged with HBSS/PBS; the pellet was resuspended in FACS buffer (PBS+2% FCS+2 mM EDTA) and stained with anti-CD45 APC antibody (BD Biosciences, Heidelberg, Germany) to deplete hematopoietic cells [24]. IECs were sorted with a MoFlow cell sorter (Cytomation, Fort Collins, CO, USA).

### RNA isolation and expression analysis

To analyze ZO-1 and ZO-2 mRNA expression in murine IECs, total RNA was isolated using the RNeasy Minikit (Qiagen, Hilden, Germany) with on-column DNase digestion using the RNase-Free DNase set (Qiagen). Isolated mRNA was reverse transcribed with 200 U Superscript II® (Invitrogen, Karlsruhe, Germany), oligo dT- and random hexamer primers (Invitrogen). PCR was performed using the following primers: ribosomal protein 9 (RPS9) mouse (mm) sense primer CTG GAC GAG GGC AAG ATG AAG C, RPS9 mm anti-sense primer TGA CGT TGG CGG ATG AGC ACA; ZO-1 mm sense primer TTT TTG ACA GGG GGA GTG G, ZO-1 mm anti-sense primer TGC TGC AGA GGT CAA AGT TCA AG; ZO-2 mm sense primer CTA GAC CCC CAG AGC CCC AGA AA, ZO-2 mm anti-sense primer TCG CAG GAG TCC ACG CAT ACA AG. Quantitative real-time RT-PCR was done with the GeneAmp 5700 Sequence Detection System (Perkin Elmer, Rodgau-Jügesheim, Germany) using Brilliant SYBR Green QPCR Core Reagent Kit (Stratagene, Heidelberg, Germany). RPS9 served as control.

### Western blot analysis

To examine the ZO-1 protein expression in IECs, lysates of sorted cells from colonized gnotobiotic mice were homogenized and subjected to sodium dodecyl sulfate-polyacrylamide gel electrophoresis (SDS–PAGE), followed by blotting the proteins on a PVDF membrane. After the blocking of unsaturated protein binding sites, the membrane was incubated with the primary antibody rabbit anti-ZO-1 (Zymed, South San Francisco, CA, USA) or rabbit anti-β actin (Sigma-Aldrich, Taufkirchen, Germany) and the secondary antibody goat anti-rabbit IgG (Dianova, Hamburg, Germany), respectively.

### Electricphysiologic measurements

The colonic mucosa was mounted between two chambers with an exposed area of 0.625 cm^2 ^and placed in an Ussing chamber. Parafilm “O” rings were used to minimize edge damage to the tissue where it was secured between the chamber halves. Tissues were bathed with HCO_3_^−^ containing solutions on both sides which were gassed with 95% O_2_/5% CO_2_. The composition (in mM) was 108 NaCl, 22 NaHCO_3_, 3 KCl, 1.3 MgSO_4_, 2 CaCl_2_, 1.5 KH_2_PO_4_, at pH 7.4. The serosal bath contained (in mM) 8.9 glucose, 10 sodium pyruvate, 10^−3^ indomethacin, and 10^−3^ tetrodotoxin; the luminal bath contained 8.9 mannitol and 10^−2^ amiloride (to block potential amiloride-sensitive Na^+^ channels). Short-circuit current (Isc), potential difference (PD) and tissue resistance (R) were recorded using the Mussler 6-channel voltage clamp system (Mussler, Aachen, Germany). ^22^Na^+^ studies were performed during voltage clamp to zero PD. 74 kBq/ml ^22^Na^+^ was added to either the serosal or the mucosal solution after reaching stable electrical parameters. After stabilization (approximately 20–30 minutes after mounting), a 45-minute period of equilibration followed, then aliquots were taken in 15-minute intervals (two intervals for basal flux, two after forskolin, and two after luminal glucose). For the presented results, we used the values from the second basal flux period, the first period after forskolin, and the first period after glucose. There were no statistically significant differences between the values obtained in the first and second flux period after forskolin and after glucose. Radioactivity was determined in a liquid scintillation counter, and bidirectional flux rates for the respective substance were calculated. The values for Isc represent the average value of the 15-minute period.

### Measurement of colonic epithelial permeability

Animals were maintained with free access to food and water. Induction of anesthesia was achieved by the administration of 10 µl/g intraperitoneal haloperidol/midazolam/fentanyl cocktail (haloperidol 12.5 mg/kg, fentanyl 0.325 mg/kg and midazolam 5 mg/kg body weight). The lower abdomen was opened by one small central incision, and a small polyethylene tube (PE100) with a distal flange was advanced to the proximal colon (immediately after the cecum), and secured by a ligature that served as inlet tube. A PE200 flanged tubing was inserted through the rectum and secured by ligature to allow for drainage through the rectum. The isolated colon segment with an intact blood supply was gently flushed and then perfused (Perfusor compact, BRAUN, Melsungen, Germany) at a rate of 30 ml/h with 150 mmol/l NaCl for 5 min, followed by perfusion with 1% Evans Blue in NaCl for 10 min. To wash-out the sticking dye in the mucus, the lumen was perfused with 6 mM acetylcysteine for 5 min followed by NaCl for 10 min. The animals were then sacrificed by cervical dislocation and the ligated colon was removed. The colon was rinsed once more with saline, its length was recorded and it was then placed in 5 ml N,N- dimethyl-formamide overnight to extract the Evans Blue. The dye concentration was measured spectrophotometrically at 620 nm (Hitachi U-2000 UV/VIS, Hitachi, Japan).

### Immunofluorescence

Tissue sections were fixed with 4% paraformaldehyde, washed extensively and blocked with porcine serum. Subsequently, the sections were incubated with the primary antibody rabbit anti-ZO-1 (Zymed) followed by incubation with a Cy3 labeled secondary goat anti-rabbit IgG antibody (Jackson Immunoresearch, Cambridgeshire, UK). The sections were then dried, covered with gelatine and visualized by fluorescence microscopy.

### Statistical analysis

Statistical analysis was performed with Origin 7.5 software (OriginLab, Northampton, MA, USA). For analysis of numeric values, the one-tailed analysis of variance and the Student's *t*-test were used. A *p*-value of <0.05 was considered significant. Error bars represent the standard error of the mean.

## Results

### ZO-1 mRNA and protein expression are elevated in gnotobiotic mice colonized with EcN

**Figure 2:**
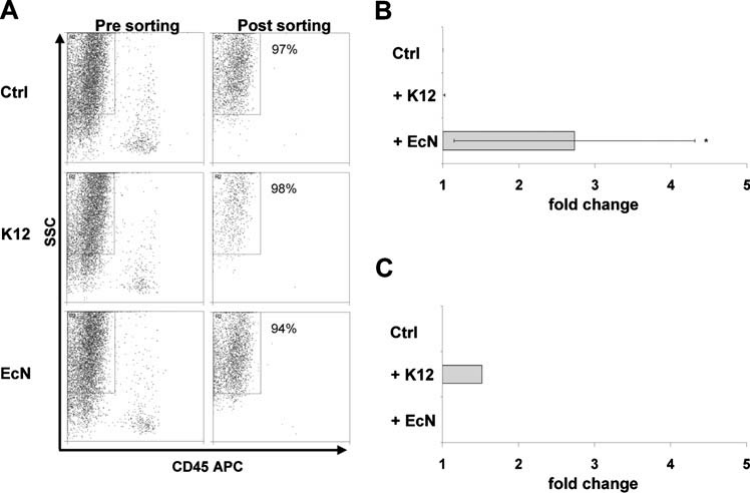
Isolation of IECs from gnotobiotic mice colonized with EcN or *E. coli* MG1655 and ZO-1 expression analysis. Gnotobiotic BALB/c mice were colonized with *E. coli* MG1655 (K12) or EcN for 6 days, respectively. Application of PBS was used as control. (A) Whole intestinal cell populations were isolated from gnotobiotic mice treated either with PBS, K12 or EcN. For sorting of a pure intestinal epithelial cell population, cells were labeled with anti-CD45 antibody to exclude hematopoietic cells and further distinguished by cell granularity and size (SSC) (Pre sorting). IECs were FACS-sorted by negative selection. Re-analysis was performed to determine the purity of sorted IECs (Post sorting). (B) Quantitative ZO-1 mRNA expression in IECs. Relative mRNA amounts were normalized with respect to expression levels of IECs from control mice (fold change = 1). Data are presented as mean of three independent experiments (n = 3/group). (C) Quantitative ZO-2 mRNA expression in IECs. Relative mRNA amounts were normalized with respect to expression levels of IECs from control mice (fold change = 1). Data are presented as mean of three independent experiments (n = 3/group). *p<0.05 EcN vs. Ctrl (relative expression values).

**Figure 3:**
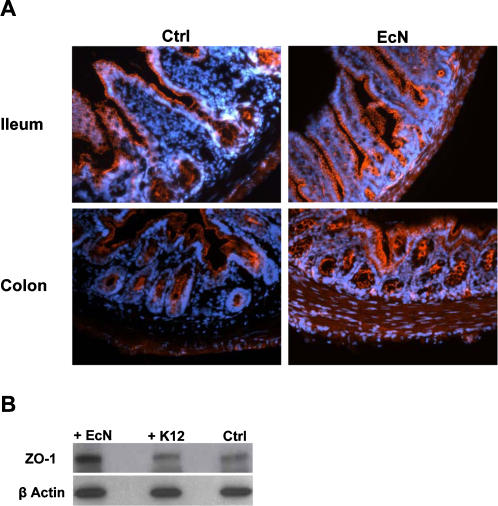
ZO-1 protein expression in ileum and colon of gnotobiotic mice. (A) Immunofluorescence staining of tissue sections from gnotobiotic control mice and mice colonized with EcN for 6 days with a fluorescent anti-ZO-1 antibody (orange). Original magnification×20. (B) Protein expression of ZO-1 in IECs. Western blot analysis of FACS sorted IECs from colonized mice was performed using anti-ZO-1 antibody. Anti-β actin antibody was used as internal control, binding to the corresponding protein with a molecular weight of 42 kDa. The ZO-1 antibody detects endogenous ZO-1 protein, displayed as a band at ∼210 kDa.

In order to investigate the impact of a single bacterial species on host IEC gene expression, we established a model for colonization of gnotobiotic mice with EcN and *E. coli* MG1655. To further investigate the *in vivo* impact of EcN on the epithelial barrier, primary IECs from the intestine of gnotobiotic mice were isolated and sorted by FACS resulting in an IEC population with greater than 94% purity (Figure 2A). ZO-1 is a TJ protein which has been described to play an important role in the prevention of intestinal barrier disruption by probiotics. Therefore, we investigated whether EcN differentially regulates ZO-1 mRNA expression *in vivo*. As depicted in figure 2B colonization of gnotobiotic mice with EcN resulted in a specific up-regulation of ZO-1 mRNA in IECs (*p*<0.05). To confirm these data at protein level, sections of ileum and colon from gnotobiotic control mice and animals colonized with EcN were stained with anti-ZO-1 antibody followed by a fluorescence-labeled secondary antibody (Figure 3A). Comparison of ZO-1 staining along the surface of the crypts revealed only a slight increase of ZO-1 protein in IECs of mice colonized with EcN. To further analyze the up-regulated ZO-1 expression at protein level, isolated IECs were investigated by Western blotting. In comparison to IECs isolated from control mice and those colonized with *E. coli* MG1655, IECs from EcN treated animals showed a markedly elevated expression of ZO-1 protein (Figure 3B). Thus, we could clearly demonstrate that colonization of gnotobiotic mice with EcN specifically up-regulates ZO-1 expression at the mRNA as well as at the protein level. Due to the important functional role of ZO-1 in the junctional complex, these findings suggest that enhancement of the intestinal epithelial barrier function by EcN could at least in part be attributed to up-regulation of ZO-1. It has also been previously demonstrated that treatment of T84 cells with EcN leads to an up-regulation of ZO-2 *in vitro* [10]. In contrast to the data generated with the T84 cell line, analysis of ZO-2 mRNA expression in IECs isolated from gnotobiotic mice colonized with EcN or *E. coli* MG1655 did not result in an increase of ZO-2 mRNA levels (fold change <2) *in vivo* (Figure 2C).

### Elevated ZO-1 expression after EcN treatment in experimental colitis

**Figure 4:**
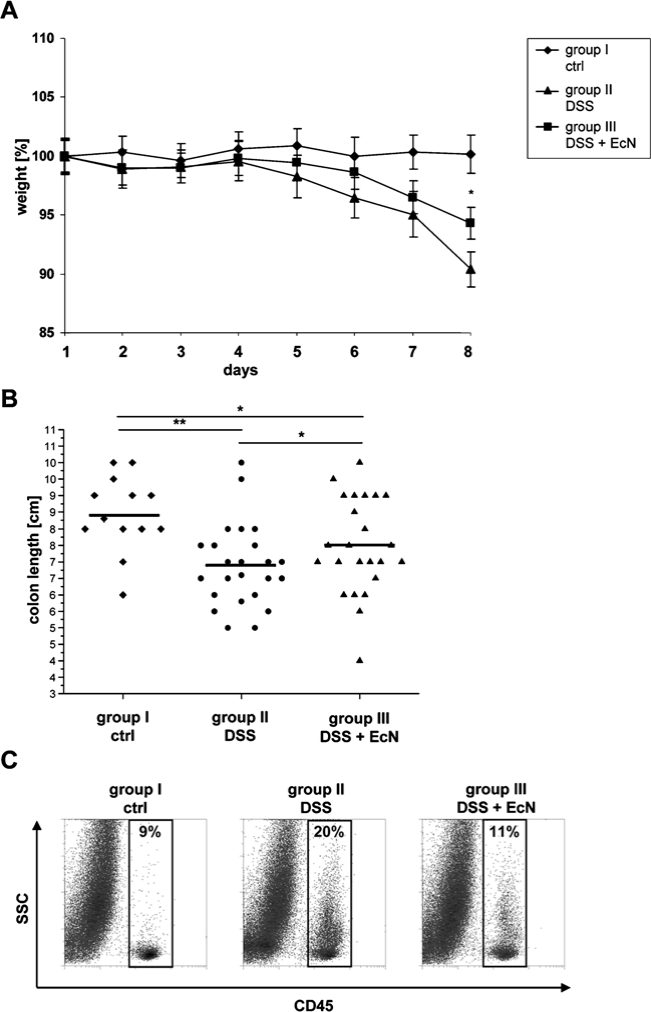
Administration of EcN in DSS induced colitis. (A) Disease severity was measured daily and is expressed in terms of body weight loss. (group II: n = 23, group III: n = 25, group I: n = 17) *p<0.05 group III vs. group II. (B) Reduction of colon length. Measurement of colon length [cm] after preparation. (group II: n = 24, group III: n = 25, group I: n = 13) *p<0.05 group III vs. group II or group III vs. group I **p<0.01 group II vs. group I. (C) Infiltration of hematopoietic cells into the colon. Whole intestinal cell populations were labeled with anti-CD45 APC antibody and measured by FACS analysis (n = 6/group).

**Figure 5:**
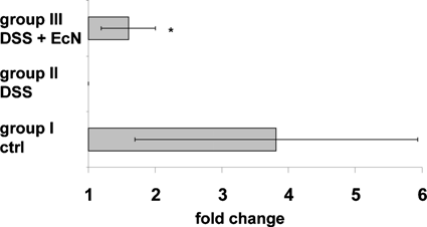
Increased ZO-1 mRNA expression in mice treated with DSS and EcN. IECs were isolated from indicated mice and relative levels of ZO-1 mRNA were normalized with respect to the expression level of IECs from DSS treated mice (fold change = 1). Data are presented as mean of four independent experiments (n = 3/group). *p<0.05 group III vs. group II (relative expression values).

It has been shown that the impaired barrier function in IBD is associated with an altered TJ structure [7], [8], [25]–[28]. Recently it was demonstrated that EcN, used as a therapeutic for the treatment of ulcerative colitis, ameliorates acute colitis in mice [29]. These observations raised the question whether an alleviated acute colitis-as a consequence of EcN treatment-may be due to its effect on the epithelial barrier. To investigate this aspect acute colitis was induced in BALB/c mice by administration of 4–6% DSS in drinking water for a period of 8 days [22]. In addition, mice were given 1.5–2×10^8^ CFU EcN or PBS orally two times a day. In contrast to untreated control mice of group I, mice exposed to DSS (group II) developed symptoms of acute colitis with diarrhea, rectal bleeding and wasting, loosing 10% of their initial body weight within 8 days (Figure 4A). Concomitant oral administration of EcN (group III) significantly ameliorated the severity of DSS-induced colitis and the loss of body weight was reduced (6%) (*p*<0.05). Healthy control mice (group I) exhibited an average colon length of 8.4 (±1.1) cm, whereas, as a consequence of severe intestinal inflammation, the colon length of DSS treated diseased mice (group II) was reduced to 6.9 (±1.2) cm (*p*<0.001). In contrast, in DSS and EcN treated mice (group III), this colon shortening was significantly attenuated with 7.5 (±1.5) cm (*p*<0.05) (Figure 4B). Colonic inflammation is correlated with strong infiltration of hematopoietic cells into the intestine. To further elaborate the beneficial effect of EcN in DSS treated mice, FACS analysis of hematopoietic cells in the colon was performed. Consistent with the reduction in loss of body weight and colon shortening, mice treated with DSS and EcN (group III) exhibited significantly lower leukocyte infiltrates in the colon in comparison to DSS treated mice (group II) (Figure 4C). To analyze whether the improved state of health after EcN treatment is accompanied with an increased ZO-1 expression, IECs were isolated from the colon of treated mice and analyzed for ZO-1 gene expression. IECs of mice treated with DSS and EcN showed elevated ZO-1 mRNA levels in comparison to DSS treated animals (*p*<0.05) (Figure 5). Although ZO-2 mRNA was not up-regulated by EcN under healthy conditions, a slight increase of ZO-2 mRNA could be detected in DSS mice treated with EcN (data not shown). These results further underline the beneficial effects of EcN on the intestinal barrier even under inflammatory conditions.

### Electrolyte transport capacity and tissue resistance after EcN treatment in experimental colitis

**Figure 6:**
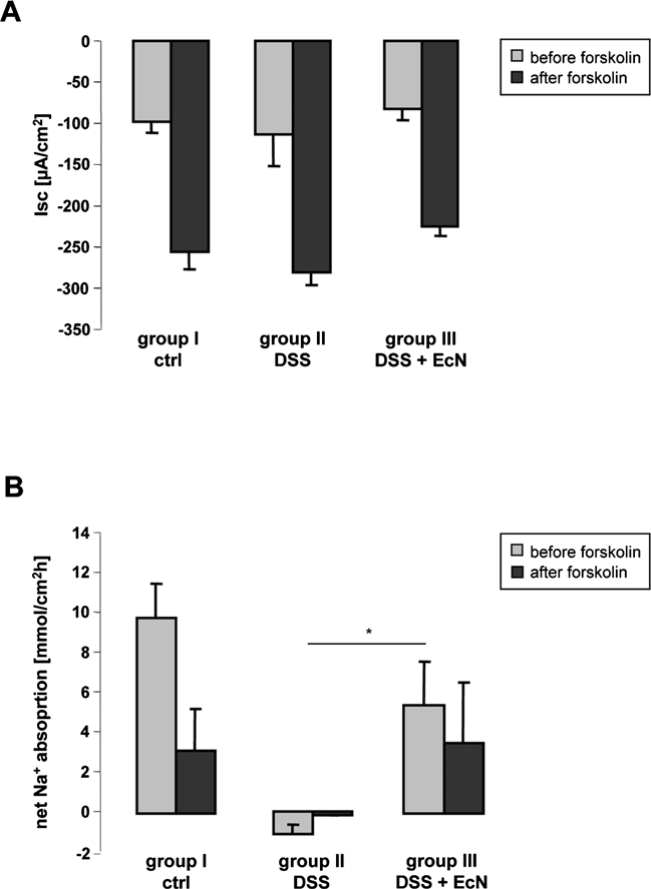
Secretory and absorptive function of isolated colonic mucosa from DSS-treated mice, with and without EcN administration, and healthy controls. (A) Basal and forskolin-stimulated Isc in DSS treated (group II n = 7), DSS+EcN (group III n = 12) and control animals (group I n = 10). The different numbers resulted from the fact that considerably more colonic segments from the DSS treated mice were so friable that they ruptured before measurements could be taken. (B) Basal and post-forskolin net Na^+^ flux rates in the three different groups. Whereas Na^+^ absorption was completely abolished in group II, active Na^+^ absorption was only partially inhibited in group III. Only in the control group I could an inhibition of Na^+^ absorption by forskolin be observed, indicating normal regulation (n = 6/group). *p<0.05

In order to assess the electrolyte transport capacity and the tissue resistance (R) in acute DSS colitis, and the influence of EcN treatment on these parameters, the basal and forskolin-stimulated Isc, basal and forskolin-inhibited Na^+^ absorption, and the tissue-resistance R in isolated colonic mucosa of inflamed DSS treated mice (group II), DSS and EcN treated mice (group III), and healthy controls (group I) were studied. To ensure that any potential inflammation-related changes would be detected, the mucosa was neither stripped nor were the prostaglandin production or neural transmission inhibited. Interestingly, no difference was found in either the basal and forskolin-stimulated Isc (Figure 6A), or in the transmucosal electrical resistance (R) between the different groups (data not shown). This demonstrates that in acute DSS colitis, the anion secretory capacity, which originates from the cryptal region of the colonic epithelium, is not perturbed. On the other hand, net Na^+^ absorption was significantly decreased in colonic mucosa of DSS and EcN treated mice (group III), and reversed to Na^+^ leakage into the luminal fluid in the mucosa of DSS treated mice (group II) (Figure 6B). Since the transporters for Na^+^ absorption are expressed in the surface colonic enterocytes, this demonstrates severe alterations in surface cell electrolyte transport following treatment with EcN.

### Reduction of colonic epithelial permeability after EcN treatment in experimental colitis

**Figure 7:**
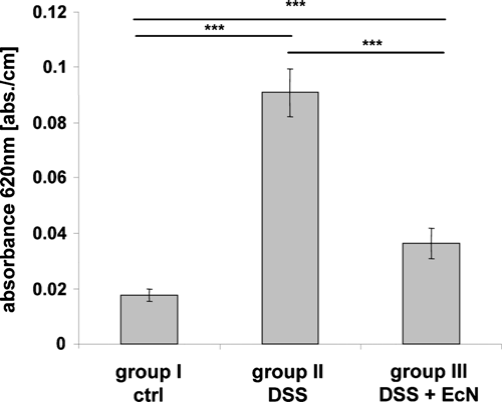
Permeability to Evans Blue in the colon of DSS treated (group II), DSS+EcN (group III) treated mice and healthy controls (group I). The graph shows a significant increase in Evans Blue uptake into the colonic mucosa of group II mice and a strong reduction in Evans Blue uptake in group III mice to almost normal values (n = 6/group). ***p<0.001

In order to address the question whether the pronounced ZO-1 expression in EcN treated mice (group III) impacts the permeability of the colonic epithelium for transport of luminal substances, the uptake of Evans Blue into the mucosa in anesthetized mice after a short-term luminal perfusion with the dye was measured. In comparison to untreated mice (group I), a strong increase of Evans Blue uptake into the colonic mucosa of DSS treated mice (group II), and a much lesser increase into the colonic mucosa of DSS and EcN treated mice (group III) was detected (Figure 7). This demonstrates that the concomitant application of EcN during colitis induction with DSS markedly ameliorates the leakiness of the colonic epithelium.

## Discussion

The current study establishes that *E. coli* Nissle 1917 positively impacts the intestinal epithelial barrier *in vivo* in three different ways. First, EcN is capable of producing a specific up-regulation of ZO-1 expression in IECs of healthy gnotobiotic mice. When treated concomitantly with EcN, IECs of mice with DSS-induced colitis also exhibit a pronounced expression of ZO-1 mRNA. Finally, EcN provides protection against the DSS-mediated leakiness of the gut in our mouse model. Our data strongly suggest that one of the protective effects of EcN treatment on colitis prevention could be a modulation of tight junctional integrity which in turn leads to preserved intestinal barrier function against noxious or infectious agents.

Critical for the development of IBDs are imbalances in mucosal immunity as well as a disturbed function of the epithelial barrier, which leads to a marked infiltration of luminal microflora [30]. Moreover, in the majority of cases, gastrointestinal diseases develop from the disruption of the intestinal epithelial barrier by enteropathogenic bacteria that alter the cellular cytoskeleton [31], [32] or affect specific tight junction proteins like ZO-1 [33]. In the past years, probiotics have been shown to be effective in the treatment of mild to moderately active IBD [18] and to reduce inflammation in animal models of colitis [29], [34]–[36]. Three large clinical trials have investigated the therapeutic efficacy of EcN in maintaining remission of UC. EcN was reported to be as efficacious as standard medication in preventing the relapse of UC [12]–[14]. Although the mechanisms leading to relapses in the pathogenesis of IBDs have not yet been clarified, there is growing evidence that increased intestinal permeability plays a key role. This report is the first to demonstrate a direct influence of the therapeutic EcN on expression of the TJ associated molecule ZO-1 *in vivo* under healthy and inflammatory conditions. We demonstrate that EcN specifically up-regulates ZO-1 expression both at the mRNA and at the protein level in IECs of gnotobiotic mice. Very recently, Zyrek *et al.* described the up-regulation of ZO-2 after EcN exposure to the T84 cell line *in vitro* [10]. However, in this study analysis of IECs from gnotobiotic mice colonized with EcN did not reveal a differential ZO-2 mRNA expression *in vivo* following EcN treatment.

In order to study functional consequences of DSS-mediated colitis and EcN treatment and also the potential significance of ZO-1 up-regulation for an enhancement of intestinal barrier function, we performed experiments aimed to assess transport and barrier function of the colonic epithelium of DSS-treated mice with and without application of EcN. To evaluate secretory and absorptive function of the colonic epithelium, we measured the basal and forskolin-stimulated Isc (which is an assessment of the electrogenic anion secretion, and its stimulation by an increase in intracellular cAMP levels), as well as net Na^+^ absorption before and after an increase in cAMP levels. Interestingly, after one week of DSS treatment, the acute colitis did not compromise the secretory function of the epithelium at all, but abolished the Na^+^ absorptive function completely. Na^+^ absorption is mediated by electroneutral (apical Na^+^/H^+^ exchangers NHE3 and possibly NHE2) and electrogenic (apical Na^+^ channel ENaC) pathways in the colon [37]. Na^+^ absorptive transporters are compromised during acute colitis, and this is one major reason for diarrhea during colonic inflammation [38].

Active fluid absorption is also dependent on intact tight junctions, otherwise a phenomenon called “leak flux diarrhea” occurs [39]–[41]. A cytokine-induced intestinal barrier dysfunction via a leak flux mechanism has recently been proposed by Schmitz et al. as potential cause for non-infectious diarrhea in HIV-infected patients, in addition to mucosal transformation with a consecutive malabsorptive mechanism [42]. When cholera toxin deletion mutants of *Vibrio cholerae* were given to healthy volunteers they still developed mild diarrhea [43]. Searching for the causative agent led to the detection of the ZOT, a toxin which causes a disruption of the tight junctions in isolated intestine [44]. Later studies revealed yet another agent from *Vibrio cholerae*, the HA/protease, that specifically interferes with the tight junction proteins occludin and ZO-1 resulting in barrier disruption [45], [46]. Thus, it became clear that the integrity of the tight junctions was necessary for the maintenance of an absorptive state of the gut epithelium. In addition, it was found that a disruption of the tight junctional complex allowed easier permeation of substances from the lumen [47]. Our experiments demonstrated a strongly elevated influx of Evans Blue into the colonic mucosa in the live mouse with DSS colitis which is indicative of increased gut permeability. Much less dye was bound to IECs of mice treated concomitantly with EcN. Combined with the absorptive Na^+^ flux in these animals, this is likely to explain the nearly normal appearance of the feces in the DSS plus EcN treated mice compared to the liquid stools of the mice with DSS colitis. It is feasible that the reduction of DSS-mediated downregulation of ZO-1 expression by EcN treatment is one reason for the enhanced barrier stability.

Several lines of evidence suggest that increased intestinal permeability has a central role in the pathogenesis of IBDs. For example, between 10–20% of presymptomatic CD patients have been shown to exhibit increased gut permeability [48], [49]. Alteration of TJ structure in UC for instance results in impaired barrier function [40]. Localization studies in mucosal biopsies of IBD patients have revealed disappearance of key TJ proteins from intercellular junctions [26], [50]. Probiotics have been shown to reduce the increased intestinal permeability *in vitro* [51] as well as in clinical trials [52]. Here we demonstrate for the first time that the substantially increased intestinal permeability of mice treated with DSS is significantly alleviated by simultaneous oral application of EcN. In addition, we observed an altered ZO-1 expression profile in IECs of mice with DSS-induced colitis. Recently, a study described the translocation of ZO-1 from the apical to the basolateral side in CD patients [53] indicating an alteration of ZO-1 under pathological conditions. Moreover, using a mouse colitis model, Resta-Lenert *et al.* have shown that the increase in intestinal permeability is associated with a decrease of occludin and ZO-1 phosphorylation [54].

However, administration of EcN in our mouse models not only diminished the clinical signs of colitis like colon shortening and weight loss, but also prevented an increase in intestinal permeability while concurrently minimizing the down-regulation of IEC ZO-1 expression. This indicates a considerable association between the severity of colitis, as evidenced by increased gut permeability, and altered ZO-1 mRNA expression levels. It can be speculated that the rise of ZO-1 expression results in a reduced intestinal permeability by an enhanced junctional complex or a reinforced interaction of the junctional complex with actin. This hypothesis is consistent with recently published data regarding the antrum mucosal protein (AMP)-18 that ameliorates DSS colitis in mice and also enhances accumulation of occludin and ZO-1 in TJ domains *in vitro* [55]. Since AMP peptide also prevented a fall in transepithelial resistance during disruption of actin filaments and stabilized the perijunctional actin during oxidant injury, it has been suggested that AMP-18 could protect the intestinal mucosal barrier by acting on specific TJ proteins and stabilizing perijunctional actin [55]. Using another murine colitis model, administration of n-3 polyunsaturated fatty acids resulted not only in reduced pathological scores but also an increase of ZO-1 protein expression [56].

The present study clearly demonstrates that EcN specifically up-regulates ZO-1 mRNA expression in IECs *in vivo*. Together with the influence of EcN on intestinal permeability and an enhanced ZO-1 expression under pathological conditions, it can be speculated that EcN augments mucosal barrier function. These *in vivo* results corroborate the *in vitro* findings from other groups by demonstrating that probiotic EcN plays an important role in the maintenance of intestinal barrier function. An improved barrier integrity elicited by EcN is an appealing explanation for the success of this probiotic in the therapy of UC and could be an important aspect in treating further human intestinal disorders, including HIV-associated diarrhea.
